# The Combined Treatment With the FLT3-Inhibitor AC220 and the Complex I Inhibitor IACS-010759 Synergistically Depletes Wt- and FLT3-Mutated Acute Myeloid Leukemia Cells

**DOI:** 10.3389/fonc.2021.686765

**Published:** 2021-08-20

**Authors:** Xiyuan Lu, Lina Han, Jonathan Busquets, Meghan Collins, Alessia Lodi, Joseph R. Marszalek, Marina Konopleva, Stefano Tiziani

**Affiliations:** ^1^Department of Nutritional Sciences, The University of Texas at Austin, Austin, TX, United States; ^2^Department of Pediatrics, Dell Medical School, The University of Texas at Austin, Austin, TX, United States; ^3^Department of Leukemia, The University of Texas MD Anderson Cancer Center, Houston, TX, United States; ^4^TRACTION - Translational Research to AdvanCe Therapeutics and Innovation in ONcology, The University of Texas MD Anderson Cancer Center, Houston, TX, United States; ^5^Department of Oncology, Dell Medical School, LiveSTRONG Cancer Institutes, The University of Texas at Austin, Austin, TX, United States

**Keywords:** metabolomics, high-throughput screening, complex I inhibitor, FLT3-inhibitor, acute myeloid leukemia

## Abstract

Acute myeloid leukemia (AML) is an aggressive hematologic malignancy with a high mortality rate and relapse risk. Although progress on the genetic and molecular understanding of this disease has been made, the standard of care has changed minimally for the past 40 years and the five-year survival rate remains poor, warranting new treatment strategies. Here, we applied a two-step screening platform consisting of a primary cell viability screening and a secondary metabolomics-based phenotypic screening to find synergistic drug combinations to treat AML. A novel synergy between the oxidative phosphorylation inhibitor IACS-010759 and the FMS-like tyrosine kinase 3 (FLT3) inhibitor AC220 (quizartinib) was discovered in AML and then validated by ATP bioluminescence and apoptosis assays. In-depth stable isotope tracer metabolic flux analysis revealed that IACS-010759 and AC220 synergistically reduced glucose and glutamine enrichment in glycolysis and the TCA cycle, leading to impaired energy production and *de novo* nucleotide biosynthesis. In summary, we identified a novel drug combination, AC220 and IACS-010759, which synergistically inhibits cell growth in AML cells due to a major disruption of cell metabolism, regardless of FLT3 mutation status.

## Introduction

Acute myeloid leukemia (AML) is characterized by the uncontrolled proliferation of immature myeloid cells within the bone marrow and blood, preventing the growth and differentiation of normal hematopoietic cells (1). In 2020, there were approximately 20,000 new cases of AML in the U.S., where it was the leading cause of death for all subtypes of leukemia (2, 3). Thus, there is an urgent need to design novel therapeutic approaches.

Owing to the ability to enhance cancer cell death, mitigate toxicity, and slow the onset of chemoresistance, combination therapy is the cornerstone of the approach to cancer treatment (4, 5). Indeed, the first-line treatment for AML is the 7 + 3 regimen that includes a combination of cytarabine and an anthracycline (doxorubicin or idarubicin) (6). However, the poor outcomes of AML patients prompt discovery of novel synergistic combinatorial treatments which is highly challenging (7). Several methods have been developed to quantify synergism in an *ex vivo* drug screening setting, such as the Bliss independence (8), Loewe (9), and highest single agent (10) models. Yet these methods are all based on single output measures, and accurate and multifaceted drug responses cannot be concluded from these kinds of homogeneous “add-mix-measure” univariate assays (11). Moreover, high attrition rates caused by poor efficacy and safety have increased the demand for secondary drug screenings that provide a molecular fingerprint to enhance the probability of success (12). At the molecular level, the highly diverse and flexible metabolism contributes to the aggressiveness of AML making it difficult to treat; for example, the diversity of fuels for energy production, including glutamine and fatty acids, and the contribution of amino acids to redox control, cell signaling, and biomass production (13). Numerous studies have found that AML cells are highly dependent on mitochondrial function for survival (14) because of their increased mitochondrial mass (15) and high oxidative phosphorylation (OXPHOS) status (13, 16). Furthermore, hypoxia, as a hallmark of the hematopoietic niche, has been shown to promote AML maintenance and progression through energetic and oxidative metabolism (17) and the negative regulation from the oncometabolite 2-hydroxyglutarate (18). These factors are not only important metabolic manifestation of AML, but also clinically relevant for identifying therapeutic interventions and targeted drug development (19, 20). Hence, we recently developed a metabolomics-based phenotypic drug screening platform (21) along with a novel algorithm to quantify synergism from multivariate datasets (22). Using a nanoelectrospray ionization direct-infusion mass spectrometry (DIMS) technique (23), we capture and fingerprint drug-induced disturbances of metabolic states in AML screening models *in vitro*.

It has been recently reported that a novel small molecule agent, IACS-010759, selectively inhibits the growth of AML cells *versus* normal hematopoietic cells *in vitro* and *in vivo* through potent inhibition of complex I of the electron transport chain and suppression of OXPHOS, modulating glutamine related downstream pathways (24–26). This study aimed to identify chemotherapeutic agents that act synergistically with IACS-010759 to kill AML cells utilizing a primary screening with a standard approach, coupled with a secondary metabolomics-based phenotypic screening. To do so, we screened a library of approved and investigational drugs in combination with IACS-010759 using an ATP bioluminescence assay to select potentially synergistic top-hits. These candidates were then evaluated by a secondary screening to examine the metabolic modulations under single and combinatorial treatments using untargeted and isotope traced metabolomics analyses. To select the top synergistic drug combinations based on observed metabolic changes, a newly established principal component analysis-based Euclidean distance synergy (PEDS) quantification algorithm was applied, from which a novel synergistic drug combination to treat AML was discovered, IACS-010759 and AC220 (22). We then further validated the synergism by ATP bioluminescence and apoptosis assays at serial doses. Further, in-depth metabolic flux analysis (MFA) revealed that the two drugs synergistically inhibit the influx of both glucose and glutamine, which impaired the energy production from central carbon metabolism, as well as pentose phosphate pathway and *de novo* nucleotide biosynthesis.

## Materials and Methods

### Materials and Reagents

Dimethyl sulfoxide (DMSO), Roswell Park Memorial Institute (RPMI) 1640, characterized and dialyzed fetal bovine serum (FBS), MSC-qualified FBS, glutamine, phosphate-buffered saline (PBS), 96-well plates, and heat-sealing foil were bought from Thermo Fisher Scientific (Waltham, MA). CellTiter-Glo 2.0 Assay kit was purchased from Promega (Madison, WI). 384-well plates (white and flat bottom, opaque) and Dulbecco’s Modified Eagle’s Medium (DMEM) were bought from Fisher Scientific (Waltham, MA). The drug library, midostaurin, FF-10101, and gilteritinib were purchased from Selleck Chemicals (Houston, TX), and tandutinib was from LC Laboratories (Woburn, MA), while the other drugs were from Cayman Chemical (Ann Arbor, MI). Filter plates (96-well, 0.45 µm PTFE) were bought from Pall Corporation (Port Washington, NY). Isotope tracers were purchased from Cambridge Isotope Laboratories (Tewksbury, MA). PCR plates (96 LoBind) were purchased from Eppendorf (Enfield, CT). APC Annexin-V (Cat. 550475) was bought from BD Biosciences (San Jose, CA). CountBright counting beads (Cat. C36950) was bought from Invitrogen (Carlsbad, CA). All solvents and chemicals for mass spectrometry were LC/MS grade from Thermo Fisher Scientific (Waltham, MA).

### Cell Models

Human cell lines, OCI-AML2, OCI-AML3, U937, MOLM-13, MOLM-14, HS-5, HS27A, and mesenchymal stem cells (MSCs) were obtained commercially from the American Type Culture Collection. HS-5, HS27A, and AML cell lines were cultured in RPMI 1640 medium, supplemented with 10% characterized FBS, and 1% 200 mM glutamine. MSCs were cultured in DMEM medium, supplemented with 10% MSC-qualified FBS and 1% 200 mM L-glutamine. All lines were maintained at 37°C in 5% CO_2_, with 21% O_2_ for normoxia condition, or 1% O_2_ for hypoxia condition.

### ATP Bioluminescence Assay

The primary high-throughput screening was conducted by measuring the relative cell viability under treatments using ATP bioluminescence assay and used as a preliminary step to the secondary screening. We performed high-throughput primary screening of a customized 284-drug Cambridge Cancer Compound Library (CCCL; Selleck Chemicals) in combination with IACS-010759 in 384-well plates on two human AML cell lines, OCI-AML3 and MOLM-13, and three human bone marrow stromal cell lines HS-5, HS-27A, and MSCs. The screening was completed in both hypoxia (1% O_2_) and normoxia conditions. For hypoxia conditions, cells were adapted to the 1% O_2_ culture environment for at least 3 days before experiments (same for the hypoxia experiments described below). AML and stromal cells were seeded at 5,000 and 1,000 cells per well, respectively. Cells were treated with 100 nM of the drug library with or without 30 nM IACS-010759, with a final concentration of 0.1% DMSO for all treatments and controls. At 24 h, ATP bioluminescence was measured by the CellTiter-Glo 2.0 Assay. Each treatment had four replicates, and the Dixon’s Q Test (27) was used to remove outliers. The Bliss index (8) for each combination was calculated from the relative cell viability of single and combo treatments.

To test synergism between FLT3 inhibitors and IACS-010759, we specifically screened 3 wild-type AML cell lines (U937, OCI-AML2, and OCI-AML3) and 2 FLT3-ITD+ AML cell lines (MOLM-13, MOLM-14) by 13 FLT3 inhibitors all currently in clinical trials (AC220, sorafenib, gilteritinib, sunitinib, ponatinib, midostaurin, ibrutinib, TP-0903, crenolanib, tandutinib, FF-10101, lestaurtinib, and KW-2449) with 10 nM IACS-010759 to test drug synergy. Cells were plated at 5,000 per well in 384-well plates and treated by serial concentrations (0.0128:5x:5000 nM) of FLT3 inhibitors with or without IACS-010759 for 72 h (two replicates each condition). The final concentration of DMSO was kept constant at 0.1%. The cell viability was tested by CellTiter-Glo 2.0 assay and Bliss indices were calculated for each concentration (28, 29).

For synergy validation between AC220 and IACS-010759, AML cell lines (U937, OCI-AML2, and OCI-AML3) were seeded into 96-well plates at 2×10^5^/mL and left untreated or treated with AC220 and/or IACS-010759 at 0.25 x, 0.5 x, 1 x, 2 x and 4 x IC_50_ doses (500 nM AC220, 10 nM IACS-010759) of each individual drug. Cell proliferation was determined by CellTiter-Glo 2.0 assay 72 h after adding drug, using standard protocols. The number of viable cells was proportional to and thus calculated based on the luminescent intensity.

### Metabolomics-Based Phenotypic Screening

Drugs selected from the primary screening proceeded to the secondary DIMS screening to test for metabolic perturbations upon different treatments in a 96-well format. OCI-AML3 cells were seeded at a density of 200,000 cells/well in fresh RPMI 1640 medium with 10% dialyzed FBS and 2 mM unlabeled or ^13^C_5_, ^15^N_2_-glutamine. Cells were treated with 100 nM of the drug library and 30 nM IACS-010759, with a final concentration of 0.1% DMSO in both normoxia and hypoxia conditions for 24 h. Each condition had at least 5 replicates. Following treatment, cells were transferred to 96-well prewashed filter plates, centrifuged at 500 g for 3 min at 4°C to remove media, and washed with 200 µL 1X PBS twice. Cells retained on filters were extracted by ice-cold 2:2:1 acetonitrile:methanol:water spiked with 14 polar internal standards (100 µL per well, extraction blanks were also included), and then vortexed at 700 rpm for 2 min. Extracted samples were collected by centrifuging the filter plates on top of 96 well PCR plates at 100 g for 1 min at 4°C. During the whole extraction, samples were kept on ice as much as possible. Aliquots of unlabeled, glutamine-labeled, and total QCs were added to each plate, and then sample PCR plates were heat-sealed for 2 s and stored at -80°C until data acquisition.

The mass spectrometer was calibrated for both positive and negative ionization modes before data acquisition as described in detail below. Samples were thawed and centrifuged at 4750 rpm 4°C for 20 min right before sample injection. Data acquisition was achieved as previously described (21) using a Q-Exactive Hybrid Quadrupole-Orbitrap Mass Spectrometer (Thermo Scientific) equipped with an automated chip-based nanoelectrospray ionization (nESI) source (Triversa NanoMate, Advion). In brief, blanks were run at the beginning and end of the whole sequence. The sample sequence for injection was randomized, with one corresponding (unlabeled or glutamine-labeled) QC sample injected every six samples, and one total QC sample injected every twelve samples. Data were acquired in both positive and negative modes (30 s acquisition time for each mode). Tandem mass spectrometry (tMS2) data was acquired on 3 replicates of unlabeled and glutamine-labeled QC samples for metabolite identification during data processing. Sample plates were kept at 4°C during acquisition.

For DIMS data analysis, raw files were converted to mzXML files by MSConvert (30) and then processed using MATLAB scripts (available to academic laboratories upon request). Features were annotated by mass accuracy (5 ppm) using the endogenous Human Metabolome Database (31), and cleaned (23) according to previously published guidelines by replicate filtering (4 out of 5 replicates), blank filtering (signal to noise ratio higher than 3), sample filtering (more than half of all samples), and missing value imputation (random forest method) (32).

### Metabolomics and Metabolic Flux Analyses

We applied ^13^C_5_, ^15^N_2_-Glutamine, and 1,2-^13^C_2_-Glucose metabolic flux analysis (MFA) to investigate the mechanism underlying IACS-010759 and AC220 synergy. AML cell lines (U937 and OCI-AML3; 10^7^ cells/sample, 3 replicates per group) were incubated with unlabeled or labeled RPMI 1640 medium. Cells were treated with 5 nM of IACS-010759 and/or 500 nM AC220, with a final concentration of 0.1% DMSO in hypoxia for 24 h. After treatment, samples were extracted as previously reported (33). In short, metabolites were extracted by 1:1 methanol:water with 10 mM ammonium bicarbonate and equal parts of chloroform. Butylated hydroxytoluene was added to the extraction buffer to preserve metabolites susceptible to oxidation. The polar fractions were spiked with a mixture of deuterated internal standards (IS) before analysis. Both polar and apolar fractions were analyzed as previously described (33) on a Q Exactive Hybrid Quadrupole-Orbitrap Mass Spectrometer equipped with an electrospray source, connected with a Vanquish UHPLC system (Thermo Scientific). Calibration of the mass spectrometer for both ionization modes corresponding to the highest m/z range was achieved with routine commercial calibration solutions provided by the manufacturer. In addition, customized calibrations were carried out at m/z 50–750 mass range as follows: for negative ionization mode, 87.00877 (pyruvic acid); 117.01624 (fumaric-d2 acid); 149.06471 (glutamic-d3 acid); 208.11399 (Tryptophan-d5 indole); 265.14790 (sodium dodecyl sulfate) and 514.288441 (sodium taurocholate); for positive ionization mode, 74.09643 (n-butylamine), 138.06619 (caffeine fragment), 195.08765 (caffeine) and 524.26496 (MRFA).

Raw files from MFA were processed using SIEVE 2.2.0 SP2 (Thermo Fisher Scientific) and a customized script that operates in the MATLAB programming environment. Metabolite identifications were achieved by matching accurate masses and retention times to an in-house mass spectral metabolite library of standards (MSMLS; IROA Technologies, Bolton, MA). A pooled QC was used to monitor instrument stability and a blank was used for background subtraction. Peaks were included in the analysis if the coefficient of variance was less than 25% in the QC replicates and signal to noise ratio was higher than 3. Probabilistic quotient normalization (34) was performed before statistical analysis.

### Apoptosis Assay

The cell lines (U937, OCI-AML2, OCI-AML3) were untreated or treated with AC220 and/or IACS-010759 at 0.25 x, 0.5 x, 1 x, 2 x and 4 x IC_50_ doses (500 nM AC220, 10 nM IACS-010759) of each individual drug. After 72 h, cells were stained with APC Annexin-V for 30 min at room temperature in the dark. The cells were then washed and resuspended in PBS with 4’,6-diamidino-2-phenylindole (DAPI). Viable cells were enumerated by using CountBright counting beads with concurrent Annexin-V and DAPI detection on a Gallios Flow Cytometer (Beckman Coulter, Indianapolis, IN). Data were analyzed by Flowjo software (Tree Star, Ashland, OR).

### Statistical Analysis

We applied the Bliss model (8) for synergism analyses, with a Bliss index higher than 0 meaning synergistic. The Student’s t-test (two-tailed) was used for significance evaluation unless described otherwise.

To quantify synergism from the metabolic datasets, we applied a recently introduced and validated novel principal component analysis (PCA)-based Euclidean Distance Synergy quantification (PEDS) algorithm able to quantify drug synergy and identify the most promising drug combinations from omics data (22). Briefly, PCA was performed on the unlabeled DIMS data for each drug combination [including the untreated control (ctrl), the individual drug treatments (drug1 and drug2), and the combination of drugs 1 and 2 (comb) groups]. The PC scores for each treatment group (p*_treatment,i_*) and the proportions of variances (*γ_i_*) on the i-th principal component (PC), obtained from PCA, were then used to calculate *PEDS* based on the following equation:

(1) $$PEDS=\frac{\sqrt[{2}]{\Sigma_{i=1}^{n}\gamma_{i}{\left(p_{comb,i}-p_{ctrl,i}\right)}^{2}}}{\sqrt[{2}]{\Sigma_{i=1}^{n}\gamma_{i}{\left(p_{drug1,i}+p_{drug2,i}-2\times p_{ctrl,i}\right)}^{2}}}$$

Any drug combination resulting in *PEDS* values higher than 1 was considered synergistic.

Data mining on in-depth MFA data was performed according to a recently published method (35). The metabolic model includes 53 reactions and 34 metabolites covering glycolysis, pentose phosphate pathway (PPP), tricarboxylic acid (TCA) cycle (oxidative and reductive), and glutaminolysis (**Table S1**). Similar to previous studies, intracellular compartments were not considered for simplification. Glucose uptake was arbitrarily set as constant 100, while other fluxes were free with a lower and upper boundary. Isotope fractions (after natural abundance correction) with standard deviations served as the data mining input (**Table S2**). All analyses were performed using mfapy 0.5.8 (https://github.com/fumiomatsuda/mfapy), where the metabolic model was used as a function to simulate the mass isotopomer distribution vector (MDV) for a given metabolite flux distribution. Using this method, the residual sum of squares between the experimental and simulated fluxes was minimized *via* the Metropolis-Hastings algorithm (36). Briefly, for each condition 8 times of 5,000,000 distributions were generated, where the initial 2,500,000 steps were discarded because of the burn-in process, and 2,500 steps were sampled from the second half every 1,000 steps. Thus, in total, a dataset of 20,000 steps was used to represent each condition. By calculating the flux ratios with a Log10 transform, metabolic reprogramming can be identified when the dataset distributions are distinct from each other between two conditions. Because of the large dataset, Cohen’s effect size was used to quantitatively measure the magnitude of the mean difference in metabolic flux ratios between two (control and treated) conditions (with an absolute number at 0.2: small effect size, 0.5: medium effect size, and 0.8: large effect size).

## Results

### Primary High-Throughput Drug Screen: Identification of Synergies Between IACS-10759 and a Drug Library

**Figure 1:**
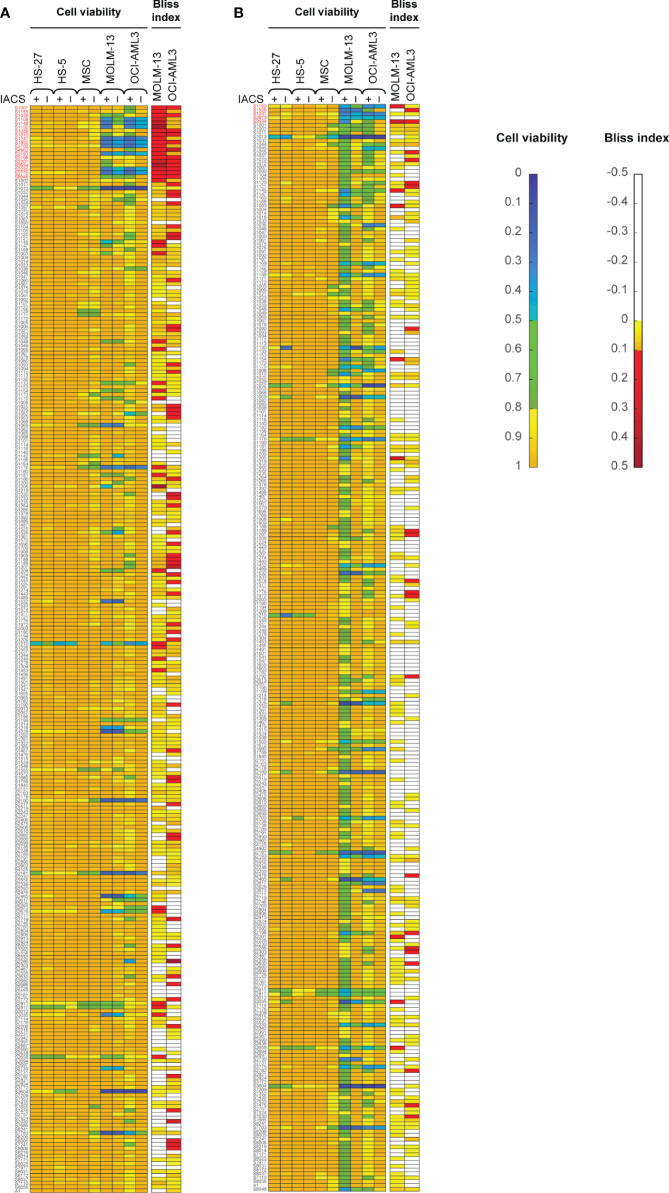
The primary drug library screening identifies 21 top-hit drug synergy candidates in leukemia and bone marrow stromal cells treated under hypoxia **(A)** or normoxia **(B)**. Bliss index [for AML cell lines; top in both **(A, B)**] and relative cell viability [for AML and bone marrow stromal cell lines; bottom in both **(A, B)**] in cells treated with 30 nM IACS-010759 and/or 100 nM drug library compounds for 24 h and in 4 replicates. Top-hit drug candidates, satisfying the primary screening criteria (relative cell viability <0.8 or Bliss Index >0.1 for the leukemia cell lines, and limited effects on the bone marrow stromal cells), are marked in red.

Here, we screened a library of 284 drugs in combination with IACS-010759 to look for potential synergistic combinations using the Bliss model. To identify drugs and drug pairs specifically effective against cancer cells rather than normal cells within the same niche, two AML cell lines (OCI-AML3 and MOLM-13) as well as three bone marrow stromal cell lines (MSC, HS-5, and HS-27A) were utilized. To mimic the *in vivo* microenvironment of leukemia, cells were tested in both normoxia and hypoxia (1% O_2_). Potential drug candidates were chosen for the secondary screening when they met both of the following criteria: (1) Relative cell viability > 0.8 for all normal cell lines under treatment with and without IACS-010759; (2) For leukemia cell lines, Bliss index > 0.1 (showing strong synergism) or relative cell viability < 0.5 under treatment with and without IACS-010759. These criteria enabled the identification of candidates that are not toxic to normal cell lines but exhibit efficacy or can be synergistic with IACS-010759 in leukemia cell lines (**Figure 1**; detailed drug names can be found in **Table S3**). Most of the drugs did not affect normal cells at a 100 nM concentration with or without 30 nM IACS-010759, while several combinatorial treatments were effective on leukemia cell lines in both hypoxia (**Figure 1A**) and normoxia (**Figure 1B**). Notably, more drugs were synergistic with IACS-010759 in hypoxia than normoxia, with 19 drugs selected *versus* only five, respectively. Interestingly, three drugs were effective in both conditions, including paclitaxel, vincristine, and rebastinib, and thus 21 (19 from hypoxia + 5 from normoxia – 3 from both) drugs in total were selected for future studies. Detailed summaries of the 21 selected drugs and their effects, including relative cell viabilities and Bliss indices, on OCI-AML3 and MOLM-13 cells are shown in **Table 1** and **Table S4**, respectively. Three out of the 21 candidates (AC220, dovitinib, and rebastinib) are known FLT3 inhibitors, and nintedanib and SGI-1776 have FLT3 as a secondary target. AC220, rebastinib, and SGI-1776 show strong synergy (Bliss index higher than 0.1) with IACS-010759 in both normoxic and hypoxic OCI-AML3 cells. Paclitaxel, rebastinib, and BMS-794833 exhibited strong synergism with IACS-010759 on MOLM-13 in both normoxia and hypoxia. Among the 3 FLT3 inhibitors, only AC220 was active against the FLT3-ITD+ MOLM-13 cells, inducing more than 55% and 70% of cell death alone in hypoxia and normoxia, respectively. This stronger nanomolar potency of AC220 compared to other FLT3 inhibitors has been reported (37). Although FLT3 inhibitors showed promising results with IACS-010759, all 21 drug candidates proceeded to the secondary screening in OCI-AML3 cells using a high-content stable isotope tracer direct infusion mass spectrometry (SIT-DIMS) analysis, for a comprehensive analysis and further selection of candidate combinations.

**Table 1:** The relative cell viability and Bliss index of OCI-AML3 treated by 21 selected drugs in combination with IACS-010759 from the primary screening in both hypoxia and normoxia conditions.

Drug ID	Drug Name	Hypoxia			Normoxia		
		o IACS-010759	w IACS-010759	Bliss Index	o IACS-010759	w IACS-010759	Bliss Index
**S1150**	Paclitaxel	0.420	0.303	0.075	0.389	0.332	0.011
**S1526**	AC220	1.101	0.855	0.134	0.982	0.741	0.128
**S1241**	Vincristine	0.324	0.216	0.074	0.334	0.289	0.006
**S2679**	Flavopiridol HCl	0.722	0.728	-0.079	0.441	0.499	-0.109
**S2634**	Rebastinib	1.022	0.815	0.104	1.053	0.797	0.133
**S1001**	Navitoclax	1.019	0.797	0.118	0.928	0.761	0.059
**S1165**	ABT-751 (E7010)	0.932	0.737	0.101	0.898	0.794	-0.001
**S1018**	Dovitinib	1.080	0.864	0.107	0.995	0.875	0.004
**S1109**	BI 2536	0.436	0.297	0.094	0.464	0.391	0.019
**S1148**	Docetaxel	0.432	0.294	0.094	0.385	0.330	0.010
**S1039**	Rapamycin	1.073	0.829	0.136	0.828	0.671	0.061
**S1010**	Nintedanib	1.129	0.787	0.228	0.983	0.803	0.066
**S1452**	Ispinesib	0.433	0.277	0.111	0.434	0.368	0.016
**S2700**	KX2-391	0.371	0.234	0.100	0.397	0.364	-0.013
**S4902**	QNZ (EVP4593)	1.004	0.522	0.381	0.917	0.772	0.038
**S2193**	GSK461364	0.462	0.316	0.100	0.477	0.405	0.017
**S2198**	SGI-1776 free base	1.086	0.851	0.125	1.025	0.798	0.108
**S2201**	BMS-794833	1.155	0.899	0.139	1.048	0.927	-0.001
**S2235**	Volasertib (BI 6727)	0.452	0.288	0.118	0.477	0.380	0.042
**S2775**	Nocodazole	0.392	0.228	0.124	0.411	0.358	0.005
**S8048**	Venetoclax	1.003	0.765	0.136	0.908	0.752	0.050

Relative cell viabilities less than 0.5 are highlighted in green, and Bliss indices higher than 0.1 are highlighted in yellow.

### The FLT3 Inhibitor AC220 Shows Synergism With IACS-010759 in a Secondary Phenotype-Based Screening

**Figure 2:**
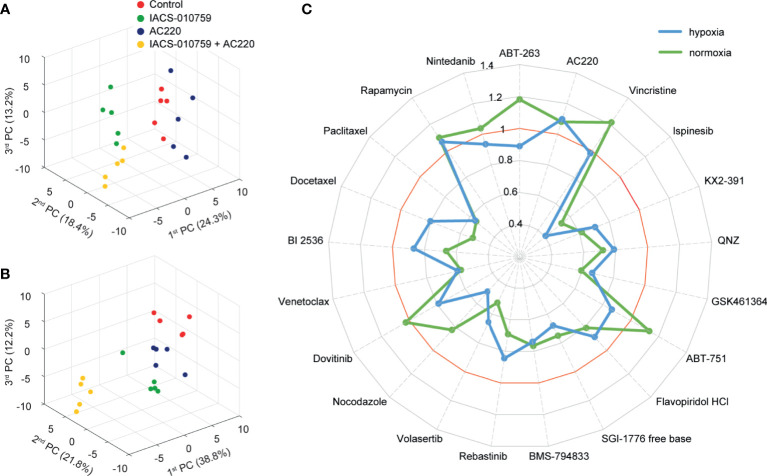
Untargeted metabolomics-based phenotypic screening by DIMS displays distinct metabolic modulations upon drug treatments and prioritizes synergistic drug combinations at the multivariate level. **(A, B)** 3D scores plots obtained from the PCA of metabolomic profiles for OCI-AML3 treated for 24 h with control, 30 nM IACS-010759 and/or 100 nM AC220 (selected from the secondary screening data as an example) in hypoxia **(A)** and normoxia **(B)**. **(C)** Radar plot of PEDS values of the 21 combinations evaluated in the DIMS secondary screening to quantify drug synergism under both hypoxia (blue) and normoxia (green) conditions after 24 h treatments.

In the secondary screening, we employed untargeted (unlabeled) and targeted (labeled) DIMS metabolomic data to identify the most promising of the 21 drug combinations selected from the primary screening. For an unbiased analysis, a total of 94 clean features (more details in methodology) were extracted out from unlabeled experiments to perform PCA. The robustness of the methodology is demonstrated by the tight clustering, showing distinct metabolomes, of samples from unique groups representative of IACS-010759 and AC220 (selected as an example) as single treatments and in combination in hypoxia (**Figure 2A**) and normoxia (**Figure 2B**).

The metabolomic profiles revealed prominent modulations at the molecular level under single and combinatorial drug treatments (**Figure S1**). Some metabolic changes are conserved in both normoxia and hypoxia conditions, such as the dramatic decrease of metabolites involved in glutathione metabolism and increase of lysophosphatidylcholines (**Figures S1A, B**: marked in red) in flavopiridol treated groups. This observation is a known consequence of the flavopiridol antitumor mechanism in breast cancer cells (38). Additionally, in majority of IACS-010759-treated cells we observed increased levels of several amino acids, including glutamine, arginine, asparagine, and lysine in both, normoxia and hypoxia (**Figures S1A, B**: marked in blue), compared to those without the complex I inhibitor. Despite this, there are distinct differences in metabolite and treatment groupings between hypoxia (**Figure S1A**) and normoxia (**Figure S1B**) conditions, and compared to the normoxia condition, hypoxia achieved a clearer separation between groups with and without IACS-010759 *via* hierarchical clustering analysis, indicating metabolic interplay with oxygen.

Following untargeted DIMS analysis, PEDS values were calculated for each combination (**Figure 2C**) to predict synergistic combinations (PEDS values greater than 1) using multivariate data. When combined with IACS-010759, AC220 and rapamycin each had a PEDS value higher than 1 in both hypoxia and normoxia, suggesting that the two combinations were synergistic. Since AC220, an FLT3 inhibitor, is currently in clinical trials for AML, we chose to investigate this combination further. Notably, the AC220/IACS-010759 (AC220-IACS) group falls into distinct sub-clusters compared to single treatment and control groups in hypoxia (**Figure S1A**). The mechanism driving synergy between IACS-010759 and AC220 can be indicated by metabolic changes among the four groups (**Figure S2**: control, AC220, IACS-010759, and AC220-IACS). In both hypoxia (**Figure S2A**: marked in red) and normoxia (**Figure S2B**: marked in red), glutathione (change dominated by IACS-010759 in hypoxia) and taurine increased to a greater extent (p<0.05 when comparing combination group with single treatment groups, except for glutathione in the hypoxia condition) in the AC220-IACS group than any single treatment or the control, indicating a compensatory response to oxidative stress induced by the combinatorial treatment. In hypoxia (**Figure S2A**: marked in blue), citrate (p<0.05 only for IACS-010759 *vs.* AC220-IACS), alpha-ketoglutarate (p<0.05), and succinate (p<0.05) from the TCA cycle, as well as glucose 6-phosphate (p<0.05 only for IACS-010759 *vs.* AC220-IACS) and pyruvate (p<0.05) from glycolysis, are the lowest in the AC220-IACS group compared to single treatment and control groups, suggesting the suppressed central carbon metabolism.

**Figure 3:**
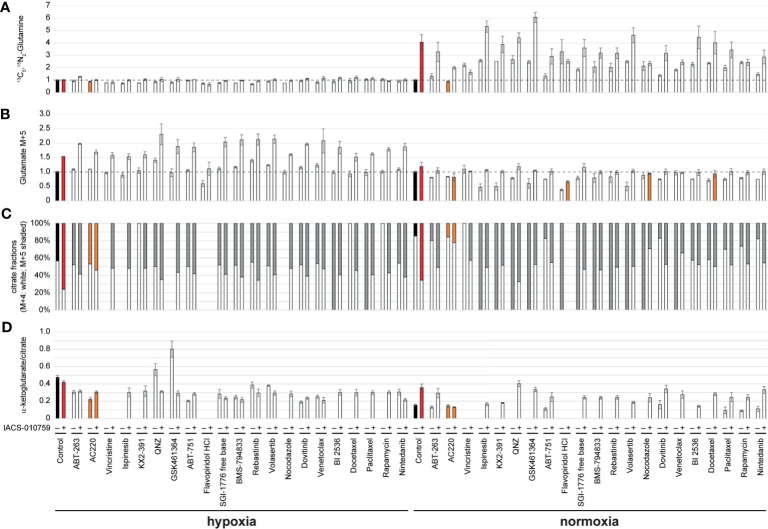
OCI-AML3 intracellular levels of representative metabolites detected by the SIT-DIMS secondary screening show extensive drug effects on cellular metabolism. OCI-AML3 intracellular **(A)** ^13^C_5_, ^15^N_2_-glutamine and **(B)** M+5 glutamate levels (normalized to control), **(C)** relative fractions of M+4 (white) and M+5 (shaded) citrate isotopes, and **(D)** alpha-ketoglutarate to citrate ratio after individual or IACS-010759-combined treatments in both hypoxia and normoxia. Treatments resulting in undetected alpha-ketoglutarate or citrate were not plotted. Shaded bars are as follows: black: control groups; red: IACS-010759-treated groups; orange: treatments specifically discussed in the results. Error bars represent standard deviations (n=5).

Given the importance of glutamine metabolism in leukemia cells and in the pathways targeted by IACS-010759, ^13^C_5_, ^15^N_2_-glutamine was used for the SIT-DIMS analysis to evaluate the 21 top hit synergistic combinations in OCI-AML3 cells in both hypoxia and normoxia conditions. This SIT-DIMS analysis from the secondary screening provided additional information on the drug mode-of-action. IACS-010759 markedly increased the uptake of ^13^C_5_, ^15^N_2_-glutamine in normoxia compared to hypoxia, together with most combination groups in normoxia as well (**Figure 3A**). In consideration of the single treatments, only AC220 decreased ^13^C_5_, ^15^N_2_-glutamine level in both normoxia (to 87.58% of control, p= 0.0159) and hypoxia (to 88.72% of control, p= 0.0012), while all combinatorial treatments in normoxia have increased ^13^C_5_, ^15^N_2_-glutamine levels. Consistent with previous reports (25), the administration of IACS-010759, either alone or in combination, enhanced glutaminolysis in both conditions (**Figures 3A, B**). However, combination with AC220, flavopiridol, nocodazole, or docetaxel in normoxia instead leads to lowered levels of glutaminolysis compared to control. In agreement with published results (25), IACS-010759, either alone or in combination, increased reductive TCA cycle activity (more pronounced in normoxia) as indicated from the citrate M5/(M4+M5) fractions together with the intensity ratio of alpha-ketoglutarate over citrate (**Figures 3C, D**). However, this trend is diminished by AC220 in the combination group. For vincristine and flavopiridol groups, alpha-ketoglutarate was undetectable both with and without IACS-010759, due to the more than 50% cell death upon these treatments. These findings indicate that AC220 inhibits the survival adaptation of AML cells upon IACS-010759 treatment, including glutamine uptake and reductive TCA cycle.

### In-Depth Investigation of Synergism Between IACS-010759 and 13 FLT3 Inhibitors

**Figure 4:**
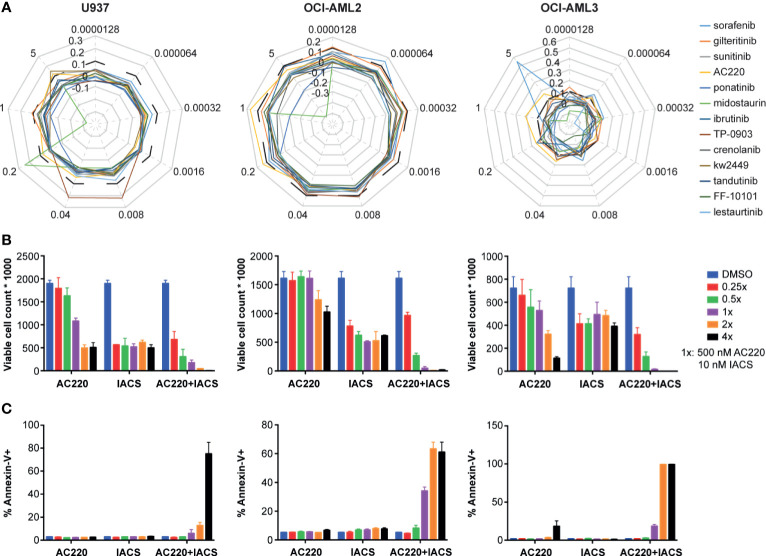
The synergy between IACS-0101759 and AC220 was validated using ATP bioluminescence assay combined with the Bliss independence model, viable cell counting, and apoptosis assay at serial doses. **(A)** Bliss index (based on Bliss independence model) for 13 FLT3 inhibitors administered in 3 leukemia cell lines (U937, OCI-AML2 and OCI-AML3) at 9 different doses (0.0000128:5x:5 µM) in combination with 10 nM IACS-010759. Bliss index values higher than 0.1 (outside of the black dashed line) represents strong synergism. **(B)** Number of viable cells and **(C)** fraction of apoptotic cells following treatment at multiple doses (0.25x, 0.5x, 1x, 2x, and 4x) of AC220 (1x: 500 nM) and/or IACS-010759 (1x: 10 nM). Error bars show standard deviations. IACS: IACS-010759.

Given that three FLT3 inhibitors showed potential synergism with IACS-010759 and that the AC220-IACS combination exhibited promising results from the secondary screening, we investigated the synergism between IACS-010759 and 13 drugs that exhibit FLT3 activity and currently in clinical trials (AC220, sorafenib, gilteritinib, sunitinib, ponatinib, midostaurin, ibrutinib, TP-0903, crenolanib, tandutinib, FF-10101, lestaurtinib, and KW-2449). To evaluate more in detail the effect of the combinations of IACS-010759 with the FLT3 inhibitors, we included in our screen a larger panel of both FLT3-wild-type and mutant AML cell lines (U937, OCI-AML2, OCI-AML3, MOLM-13, and MOLM-14) than in our initial screen. Normal cells were generally not affected by FLT3 inhibitors in our initial screen and were not considered for this analysis. The Bliss index for each concentration was calculated and plotted for different cell types (**Figure 4A**: U937, OCI-AML2, and OCI-AML3, **Figure S3**: MOLM-13, and MOLM-14). Among the 13 FLT3 inhibitors, only AC220-IACS had a Bliss index higher than 0.1 at ideal concentration windows across multiple cell types (wild-type U937, OCI-AML2, OCI-AML3, and mutant-type MOLM-13). The synergism between AC220 and IACS-010759 on U937, OCI-AML2, and OCI-AML3, was also validated using serial doses of IACS-010759 and AC220 by ATP bioluminescence (**Figure 4B**) and apoptosis assays (**Figure 4C**).

### IACS-010759 and AC220 Synergistically Impaired Both Glutamine and Glucose Influx

We then further investigated the metabolic modulation associated with the combined IACS-010759 and AC220 treatment using in-depth metabolic flux analysis (MFA) in two of the wild-type AML cell lines showing strong synergism in the secondary screen (U937 and OCI-AML3). The experiment was conducted under hypoxic condition, mimicking the physiologic conditions of the bone marrow and known to confer the chemoresistance (39). Because glucose and glutamine utilization were implicated as drivers of the AC220-IACS synergy, ^13^C_5_, ^15^N_2_-glutamine, and 1,2-^13^C_2_-glucose were used as separate tracers. Leukemia cells were incubated with the unlabeled/labeled medium for 24h and concurrently treated with IACS-010759 and/or AC220. IACS-010759 concentration of 5 nM was chosen because of its clinical relevance, and the AC220 concentration was set at 500 nM, based on the optimal synergy achieved from a dose ratio of AC220/IACS-010759 at around 100 (**Figure 4A**).

**Figure 5:**
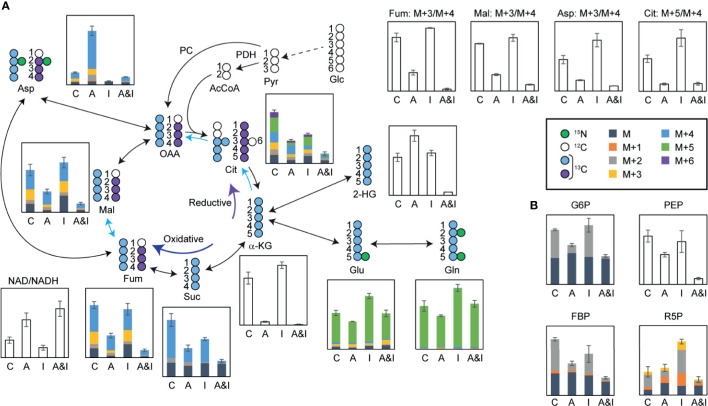
In-depth metabolic flux analysis and data mining reveal that extensive disruption of cells metabolism following treatment contributes to the synergy between IACS-010759 and AC220. U937 cells were treated with 500 nM AC220 and/or 5 nM IACS-010759 for 24 h. Relative metabolite levels are overlayed on a schematic representation of the flux of isotopically labeled **(A)** glutamine (^13^C_5_, ^15^N_2_-glutamine) through the TCA cycle or **(B)** 1,2-^13^C_2_-glucose through glycolysis and pentose phosphate pathway. Blue arrows highlighting specific TCA cycle reactions indicate metabolic reactions with Cohen’s d-values lower than -0.8. Shaded bars show ^13^C enrichment fractions; empty bars show total pool intensities or total pool intensity ratios, or isotope intensity ratios (average of three replicates). Error bars represent standard deviations (n=3). C, control; A, AC220; I, IACS-010759; A&I, AC220+IACS-010759; Glc, glucose; Pyr, pyruvate; PDH, pyruvate dehydrogenase; AcCoA, acetyl CoA; PC, pyruvate carboxylase; OAA, oxaloacetate; Cit, citrate; α-KG, alpha-ketoglutarate; Glu, glutamate; Gln, glutamine; Suc, succinate; Fum, fumarate; Mal, malate; Asp, aspartate; NAD, oxidized form of nicotinamide adenine dinucleotide; NADH, reduced form of nicotinamide adenine dinucleotide; G6P, glucose 6-phosphate; PEP, phosphoenolpyruvate; FBP, fructose 1,6-bisphosphate; R5P, ribose 5-phosphate.

While both of the individual treatments modulated glutamine consumption and TCA cycle dynamics, the most dramatic effects on the TCA cycle were observed following the combined treatment. Significant reductions in the levels of TCA cycle metabolites (**Figure 5A**)—including alpha-ketoglutarate, succinate, fumarate, malate, and citrate—pointed to reduced mitochondrial activity following the combined treatment, which was validated by an increased ratio of oxidized/reduced forms of nicotinamide adenine dinucleotide (NAD/NADH). Interestingly, the total pool of the 2-hydroxyglutarate, an oncometabolite associated with tumorigenesis (40), which increased following the individual treatments, was significantly reduced in response to the combined treatment. Significant decreases in the citrate M+5/M+4 ratio, and the fumarate, malate, and aspartate M+3/M+4 ratios demonstrate the suppression of the reductive TCA cycle by the combinatorial treatment, which validated the SIT-DIMS result. Isotopomer analysis by data mining method using mfapy (35) further supported TCA cycle suppression by AC220-IACS. Flux ratios were calculated by dividing each reaction within the chosen metabolic model (see methods) by the reaction catalyzed by citrate synthase, which lies at the intersection between glycolysis and TCA cycle, enabling evaluation of the contributions of different fluxes to citrate biosynthesis. Fluxes with large effect sizes between combination and control groups were marked (**Figure 5A**: blue means decreased flux in combination). Significantly smaller contributions from isocitrate dehydrogenase, ATP citrate lyase, and fumarate hydratase were observed in the combination treatment group, again proving the inhibition of the oxidative and reductive TCA cycle. Detailed results of this isotopomer analysis are shown in **Table S5**.

In addition to the impact on the TCA cycle, significant reductions in the absolute concentration and 1,2-^13^C_2_-glucose-derived fractions of glucose 6-phosphate, fructose bisphosphate, phosphoenolpyruvate, and ribose 5-phosphate in samples treated with AC220 (alone or in combination) point to impaired glycolysis and pentose phosphate pathway, an effect that is more pronounced in the combined treatment (**Figure 5B**). Consequently, AC220 results in suppressed *de novo* nucleotide biosynthesis, as shown by the decreased total pool (**Figure S4A**) of nucleotides and labeling incorporations from glucose (**Figure S4B**) and glutamine (**Figure S4C**). We also investigated the *de novo* synthesis of Coenzyme Q (CoQ), an essential electron carrier in the electron transport chain. Total pools of CoQ_10_ and CoQ_10_H_2_ were depleted significantly by AC220, with acetyl CoA showing similar trends (**Figure S5A**). Glutamine labeling data demonstrated that this depletion was caused by the suppression of glutamine incorporation by AC220, which is also more pronounced in the combination group (**Figure S5B**). The slightly decreased CoQ_10_H_2_/CoQ_10_ ratio (**Figure S5A**), together with a significantly increased NAD/NADH ratio (**Figure 5A**) in the combinatorial treatment group, suggests that electron deficiency leads to the lowered energy production as supported by the ATP bioluminescence data. Similar results were observed in OCI-AML3 cells (**Figure S6**).

Overall, the combinatorial treatment with IACS-010759 and AC220 impaired AML cell metabolism tremendously, and to a much greater extent than any of the individual treatments alone. Influx inhibition of the two main carbon sources, glucose and glutamine, was shown to impair the TCA cycle and glycolysis for energy production, as well as the pentose phosphate pathway and *de novo* nucleotide biosynthesis.

## Discussion

The enormous molecular heterogeneity of AML is a major roadblock to developing effective treatments. Exploiting the vulnerabilities introduced by aberrant genetic and metabolic alterations is essential to improving current therapeutic strategies. Currently, AML prognoses vary widely depending on the presence or absence of recurrent gene mutations, such as those affecting FLT3, which occur in 30% of AML patients and are associated with poor clinical outcomes (41). In contrast, a program of increased glucose and glutamine uptake to support energetic and anabolic demands (42–44) is a defining characteristic of most AML cells. Extensive work shows that AML cell metabolism is also highly plastic, enabling them to invade and flourish within distinct microenvironments of the bone marrow (45). Hypoxia, a hallmark of the hematopoietic niche, favors leukemia progression and relapse compared to normoxia (46), reflecting altered metabolism between the two conditions. In the current study, we employed a comprehensive metabolomics-based phenotypic screening platform to identify synergistic drug combinations for selectively targeting AML cell types with consideration for the above genetic and phenotypic differences. This effort successfully identified an FLT3 inhibitor, AC220, that effectively synergizes with an OXPHOS inhibitor, IACS-010759, to deplete AML cells, and importantly this phenomenon was not limited to FLT3 mutant types.

Along with midostaurin and gilteritinib being recently approved for the treatment of FLT3-mutated AML patients, multiple FLT3 inhibitors are in preclinical development or clinical trials (47). Evidence from clinical trials also suggests that adding FLT3 inhibitors to frontline intensive chemotherapy improves survival in patients with AML (48). AC220 is a highly selective nanomolar potency inhibitor of FLT3 which also inhibits c-kit (49). Of interest, AC220 showed activity also in FLT3 wild-type AML, with composite complete remission responses of about 30%, in the initial trials in relapsed/refractory AML patients (50, 51); the QUANTUM-First frontline randomized phase III multinational study of induction therapy (3 + 7) with AC220 *versus* placebo in frontline FLT3-ITD AML patients completed enrollment late 2020 and final results are expected late 2021 (NCT02668653). Thus, we investigated synergism between 13 FLT3 inhibitors currently in clinical trials and IACS-010759 as measured by Bliss indices across multiple doses. We found that only AC220 showed strong synergism with IACS-010759, which may be due to the unique anti-kit activity of AC220, and thus requires additional studies on the molecular mechanisms. The only other combination selected from secondary screening, rapamycin and IACS-010759, was not validated in our study, however, studies have found that IACS-010759 inhibits cell growth by inducing mammalian Target of Rapamycin (mTOR) suppression in sensitive AML cells (26). An mTOR inhibitor itself, rapamycin might assemble a more prominent cytotoxic effect on AML when combined with IACS-010759.

The secondary screening further investigated metabolic perturbations induced by single and combinatorial drug treatments under both hypoxia and normoxia conditions. It demonstrated that IACS-010759 suppressed aspartate synthesis and increased glutamine utilization dramatically in both normoxia and hypoxia, in agreement with previous studies conducted in normoxia ^18^. Differences in the impact of IACS-010759 on TCA cycle intermediates were observed between normoxia and hypoxia, which can be resolved using the stable isotope tracer analysis to distinguish modulation of glucose and glutamine utilization. Although the tested FLT3 inhibitors (AC220, dovitinib, nintedanib, SGI-1776, and rebastinib) share similar signal transduction targets, they induced distinct metabolic profiles with or without IACS-010759, demonstrating the sensitivity of the metabolomic monitoring approach (52). Metabolomics studies (53–55) have been focused on the FLT3-mutant cells treated by FLT3 inhibitors, but here we demonstrated the effectiveness of AC220, particularly in combination with IACS-010759, on wild-type AML cells. In agreement with previous results (53), AC220 impairs glycolysis significantly with decreased glucose-6-phosphate and pyruvate, an effect that is enhanced when combined with IACS-010759. In spite of the reported conflicting effects on glutaminolysis by AC220 in the FLT3-mutant cell types (53, 54), glutamine utilization and the TCA cycle were significantly inhibited in wild-type OCI-AML3 cells by AC220 with and without IACS-010759 as determined by both untargeted and isotope traced screening data.

Another notable aspect of this work is that the synergy mechanism derived from the screening data is validated by a comprehensive metabolic flux analysis, enabling us to show that AC220 and IACS-010759 synergistically impair glycolysis and TCA cycle with the reduced influx of both glutamine and glucose. Although, consistent with published results (24), single treatment by IACS-010759 decreased the NAD/NADH ratio demonstrating the inhibition of complex I from the electron transport chain, the combinatorial treatment increased NAD/NADH significantly indicating the increased reactive oxygen species production and inhibited alpha-ketoglutarate dehydrogenase (56). Together with the decreased CoQ_10_H_2_/CoQ_10_ ratio, the increased NAD/NADH further support the shortage of electrons, in line with the suppression of both glycolysis and TCA cycle, for energy supply of AML cells.

Taken together, our findings demonstrate that high-throughput and high-content metabolomics approaches by SIT-DIMS can be used to identify previously unknown synergy between two or more candidate agents at the screening level. Although the metabolic profiling by DIMS is less in-depth than the traditional chromatography approaches using multiple columns, it can capture the dominant variables that contribute the most to the clustering of samples (57), and thus monitor drug responses and rapidly evaluate novel combinatorial therapeutic strategies. Notably, DIMS-based secondary screen can be easily generalized for use with any cellular model system and the PEDS approach is appropriate to evaluate synergy based on to any type of –omics data. Unlike simple live/dead screens, the metabolomics data associated with compounds are reusable for the discovery and design of novel relationships between compounds. Nevertheless, further investigation of the IACS-010759 and AC220 combination in treating AML is warranted.

## Data Availability Statement

The original contributions presented in the study are included in the article/**Supplementary Material**. Further inquiries can be directed to the corresponding authors.

## Author Contributions

All authors contributed to data acquisition, analysis, drafting, or revising the article. All authors contributed to the article and approved the submitted version.

## Funding

The authors received the following financial support for the research, authorship, and/or publication of this article: This research is in part supported by the MD Anderson Cancer Center Leukemia SPORE P50 CA100632, R01 R01 CA206210, and CPRIT RP180309.

## Conflict of Interest

The authors declare that the research was conducted in the absence of any commercial or financial relationships that could be construed as a potential conflict of interest.

## Publisher’s Note

All claims expressed in this article are solely those of the authors and do not necessarily represent those of their affiliated organizations, or those of the publisher, the editors and the reviewers. Any product that may be evaluated in this article, or claim that may be made by its manufacturer, is not guaranteed or endorsed by the publisher.
