# Supplementary material for: The Combined Treatment With the FLT3-Inhibitor AC220 and the Complex I Inhibitor IACS-010759 Synergistically Depletes Wt- and FLT3-Mutated Acute Myeloid Leukemia Cells
Source: Front Oncol. 2021 Aug 20;11:686765. doi: 10.3389/fonc.2021.686765 (PMC8417744; doi:10.3389/fonc.2021.686765)
Supplement: Supplementary file 1 [file DataSheet_1.zip › Supplementary Figures.docx]

**Supplementary Information**

The combined treatment with the FLT3-inhibitor AC220 and the complex I inhibitor IACS-010759 synergistically depletes wt- and FLT3-mutated acute myeloid leukemia cells

Xiyuan Lu ^1,2^, Lina Han ^3^, Jonathan Busquets ^1,2^, Meghan Collins ^1,2^, Alessia Lodi ^1,2^, Joe Marszalek ^4,5^, Marina Konopleva ^3*^, Stefano Tiziani ^1,2,6*^

^1^Department of Nutritional Sciences, The University of Texas at Austin, Austin, TX, USA

^2^Department of Pediatrics, Dell Medical School, The University of Texas at Austin, Austin, TX, USA

^3^Department of Leukemia, The University of Texas MD Anderson Cancer Center, Houston, TX, USA

^4^Center for Co-Clinical Trials, The University of Texas MD Anderson Cancer Center, Houston, TX, USA

^5^Institute for Applied Cancer Science, The University of Texas MD Anderson Cancer Center, Houston, TX, USA

^6^Department of Oncology, Dell Medical School, LiveSTRONG Cancer Institutes, The University of Texas at Austin, Austin, TX, USA

*** Correspondence:**Marina Konopleva, M.D, PhD.

mkonople@mdanderson.org

Stefano Tiziani, Ph.D.
tiziani@austin.utexas.edu


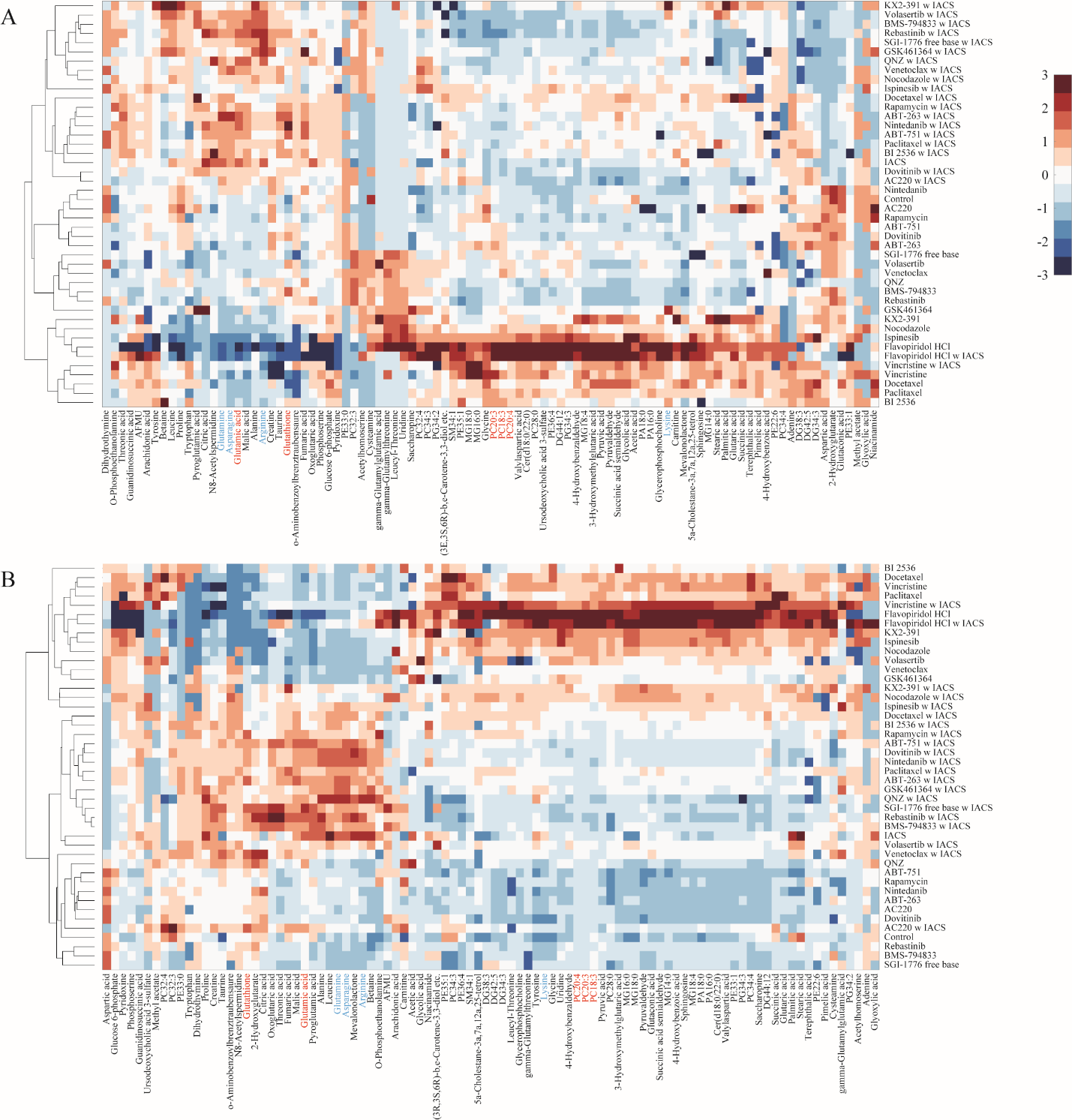


**Supplementary Figure 1.** Metabolic heatmaps of OCI-AML3 under different treatments in both hypoxia and normoxia from secondary screening.

Heatmaps of metabolic intensities detected by DIMS in OCI-AML3 treated by 30 nM IACS-010759 and/or 100 nM top candidates from primary screening for 24 h in hypoxia (**A**) and normoxia (**B**); x axis shows metabolites; y axis shows different treatments. Metabolites are clustered by Euclidean distance, and treatment conditions are clustered by Spearman’s correlation. Intensities are normalized across different treatments for each metabolite, color-coded by red to white to blue color showing decreasing trend.

IACS: IACS-010759; w: with


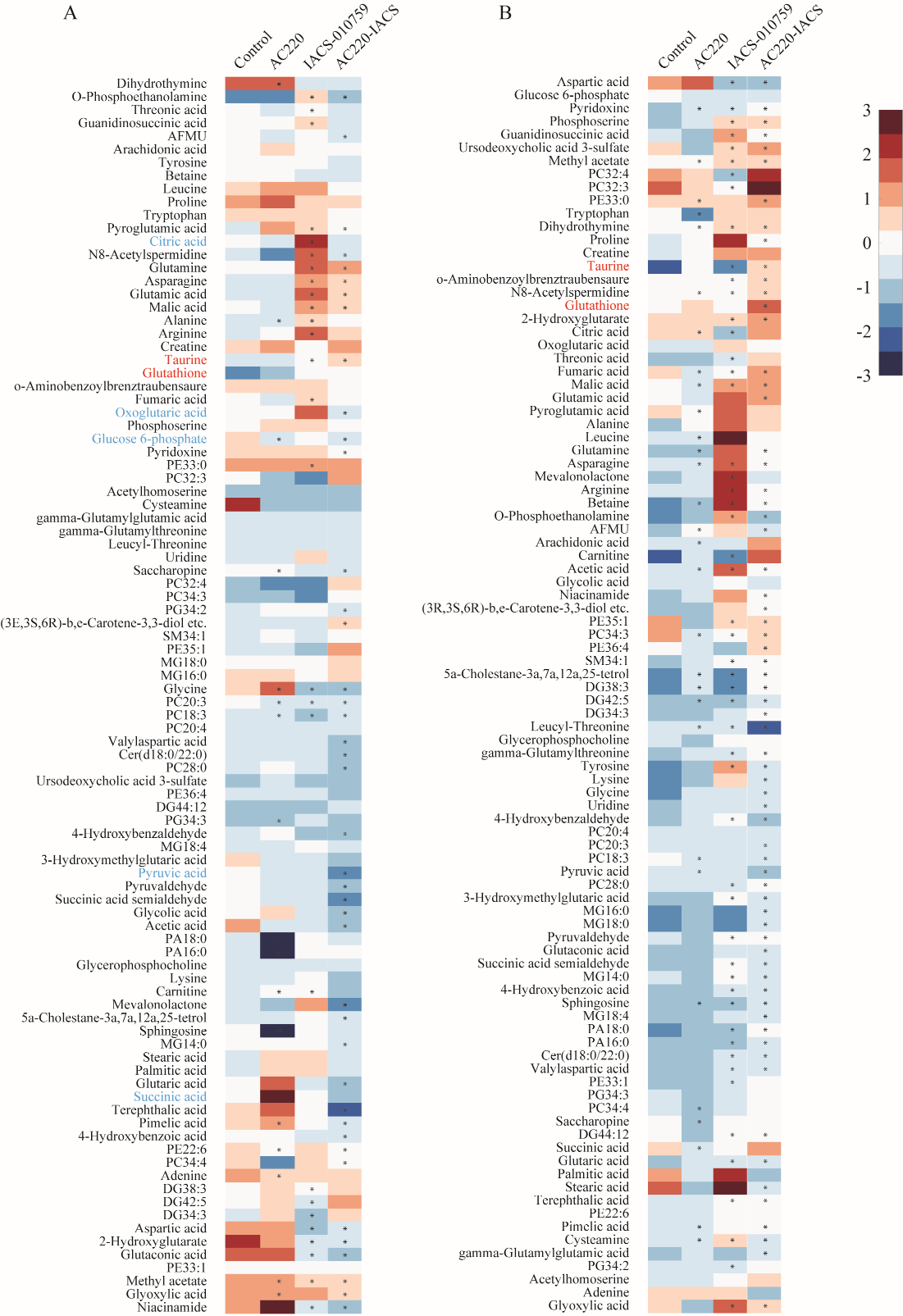


**Supplementary Figure 2.** Metabolic heatmaps of OCI-AML3 under IACS-010759 and/or AC220 treatments in both hypoxia and normoxia from secondary screening.

Cropped regions from **Fig. S1** showing heatmaps of metabolic intensities detected by DIMS in OCI-AML3 treated by 30 nM IACS-010759 and/or 100 nM AC220 for 24 h in hypoxia (**A**) and normoxia (**B**); x axis shows metabolites; y axis shows different treatments. Intensities are color-coded by red to white to blue color showing decreasing trend. Metabolites changed significantly (p < 0.05) by the Student’s t-test (two-tailed) compared to the control group were marked as “*” in the heatmap.

**
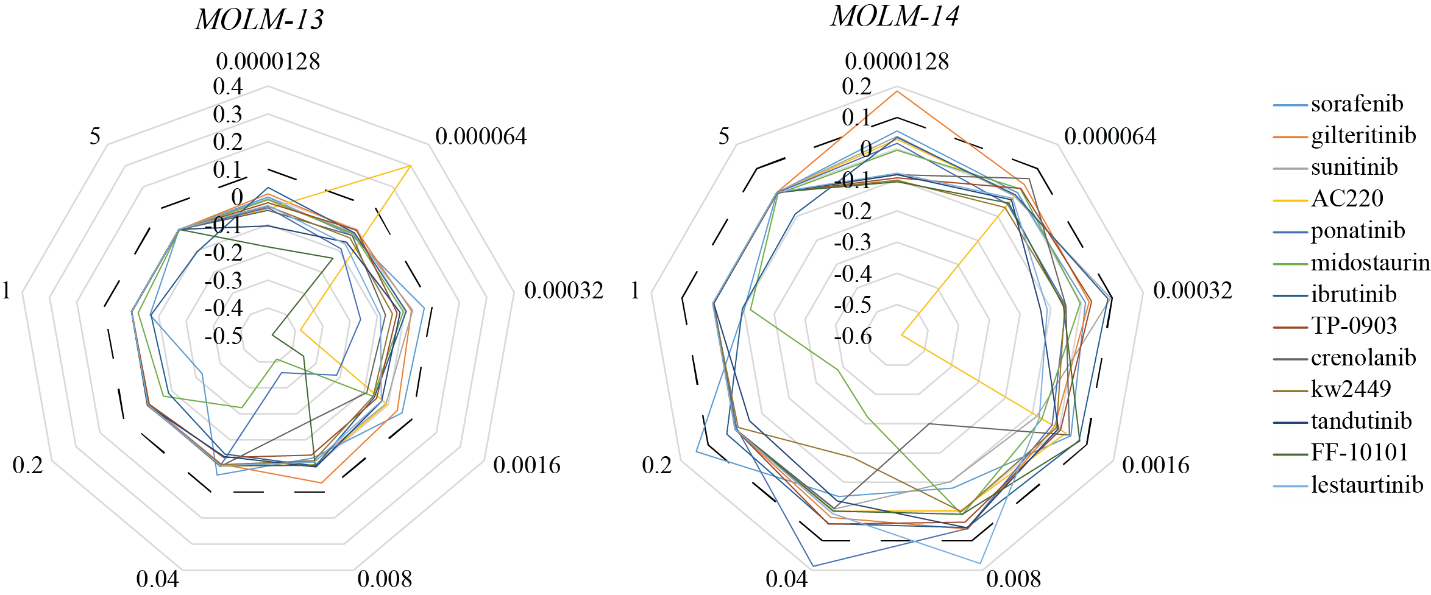
**

**Supplementary Figure 3.** Synergism between 13 FLT3 inhibitors and IACS-010759 on MOLM-13 and MOLM-14 was evaluated by Bliss independence model at serial doses.

Radar plots of bliss indices calculated from relative cell viabilities after single and combinatorial treatments by Bliss independence model. Thirteen FLT3 inhibitors at serial doses (0.0128:5x:5000 nM) were evaluated on the synergism with IACS-010759 (10 nM). Bliss index at 0.1 are highlighted in black, higher than which represents for strong synergism.


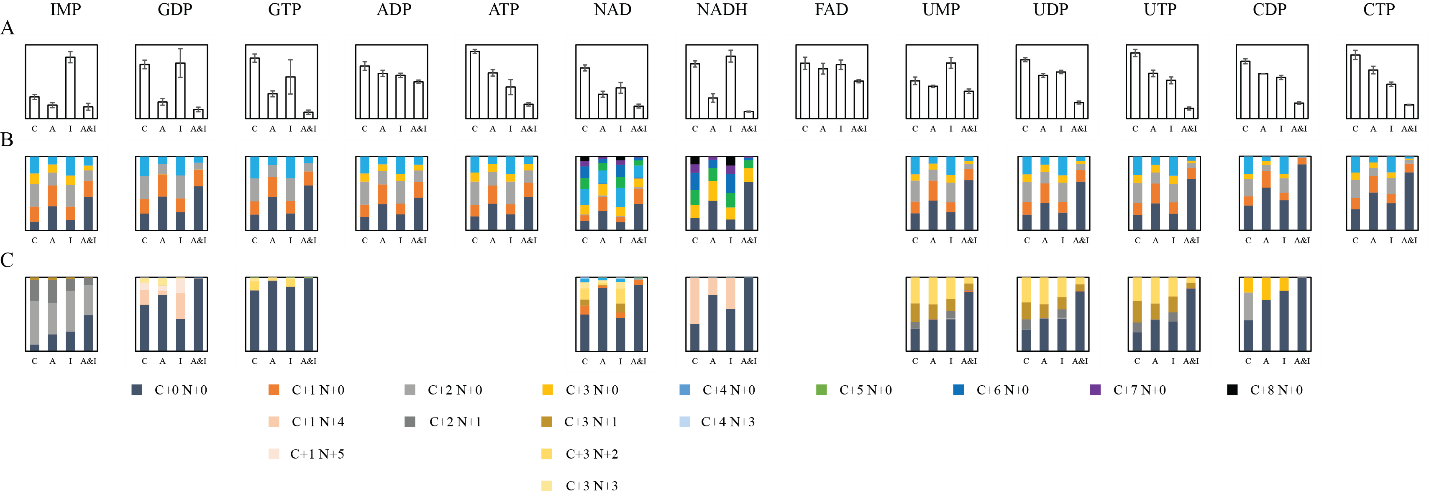


**Supplementary Figure 4.** Bar plots of total pool, 1,2-^13^C_2_-Glucose, and ^13^C_5_, ^15^N_2_-Glutamine labeling incorporation of nucleotides in U937 after treatments.

U937 cells were treated by 500 nM AC220 and/or 5 nM IACS-010759 with 1,2-^13^C_2_-Glucose or ^13^C_5_, ^15^N_2_-glutamine labeling for 24 h. (**A**) Total pool of nucleotide intensities (average of three replicates); error bars represent for standard deviations of total pool intensities of each metabolite (n=3). (**B**, **C**) Schematic representation of the flux of isotopically labeled glucose (**B**) and glutamine (**C**) through nucleotide synthesis; color-coded bars are showing ^13^C and/or ^15^N incorporations (fractions of different isotopes for each metabolite).

C: control; A: AC220; I: IACS-010759; A&I: AC220-IACS; IMP: inosine monophosphate; GDP: guanosine diphosphate; GTP: guanosine triphosphate; ADP: adenosine diphosphate; ATP: adenosine triphosphate; NAD: oxidized form of nicotinamide adenine dinucleotide; NADH: reduced form of nicotinamide adenine dinucleotide; FAD: oxidized form of flavin adenine dinucleotide; UMP: uridine monophosphate; UDP: uridine diphosphate; UTP: uridine triphosphate; CDP: cytidine diphosphate; CTP: cytidine triphosphate.

**
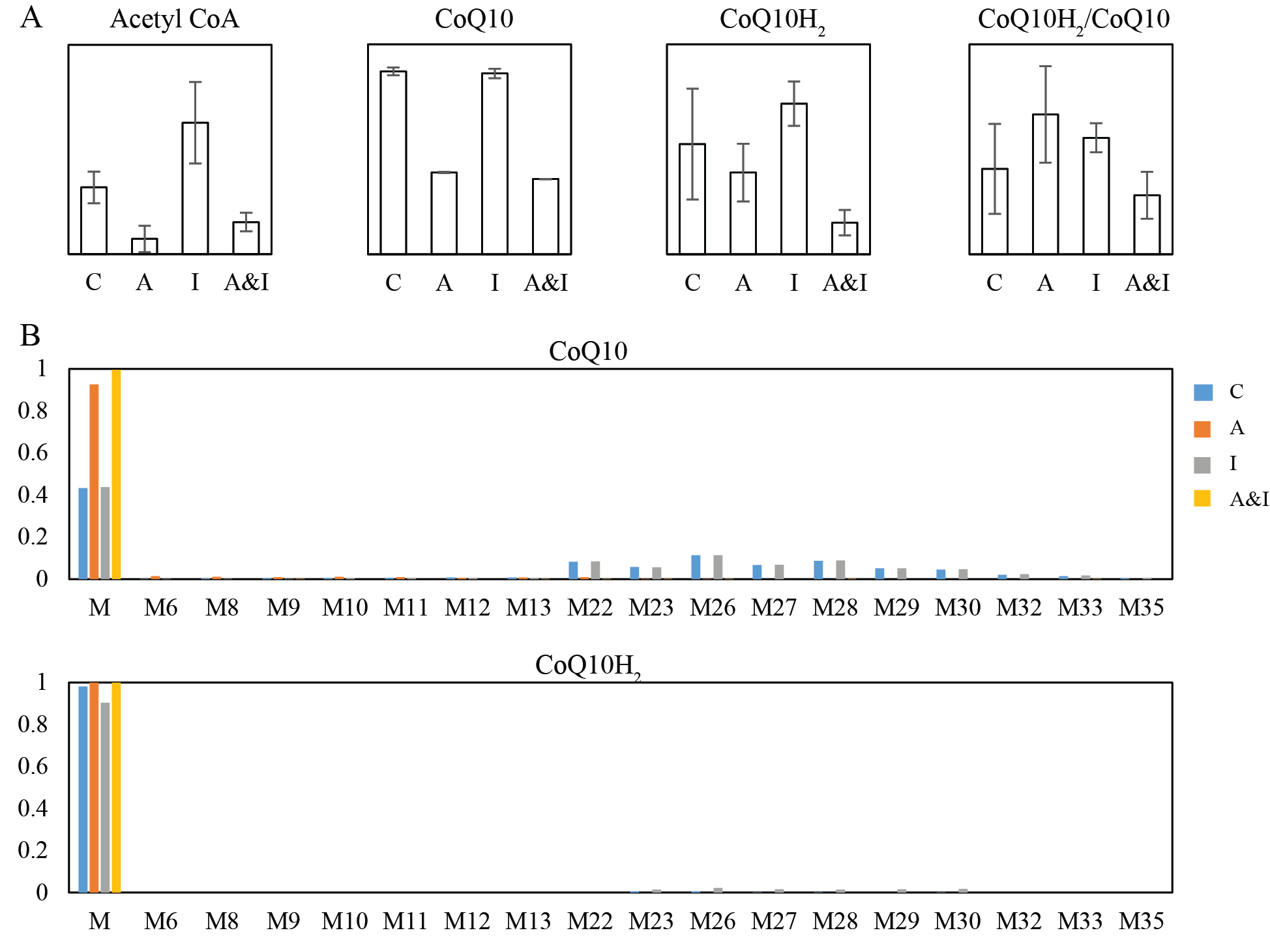
Supplementary Figure 5.** Bar plots of total pool, and ^13^C_5_, ^15^N_2_-Glutamine labeling incorporation of metabolites from Coenzyme Q biosynthesis pathway in U937 after treatments.

U937 were treated by 500 nM AC220 and/or 5 nM IACS-010759 with ^13^C_5_, ^15^N_2_-glutamine labeling for 24 h. (**A**) Total pool (ratio) of nucleotide intensities (average of three replicates); error bars represent for standard deviations (n=3). (**B**) Schematic representation of the flux of isotopically labeled glutamine through oxidized and reduced form of Coenzyme Q10; color-coded bars are showing fractions of different isotopes for each metabolite in different treatment groups (control: blue; AC220: orange; IACS-010759: gray; AC220-IACS: yellow).

C: control; A: AC220; I: IACS-010759; A&I: AC220-IACS


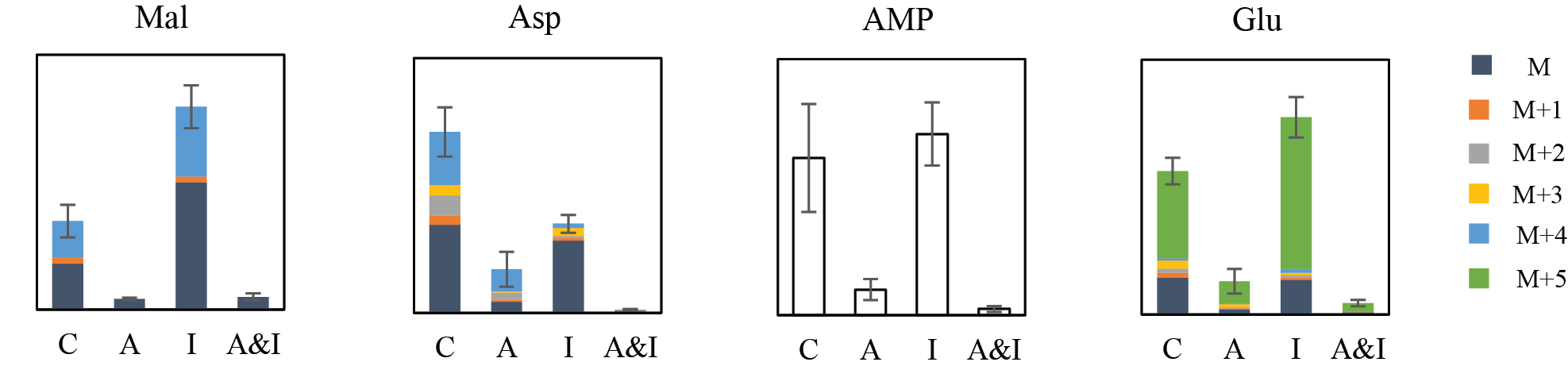


**Supplementary Figure 6.** Bar plots of ^13^C_5_, ^15^N_2_-Glutamine labeling incorporation of representative metabolites in OCI-AML3 after treatments.

Treatment-induced changes in OCI-AML3 intracellular glutamine flux. OCI-AML3 were treated by 500 nM AC220 and/or 5 nM IACS-010759 with ^13^C_5_, ^15^N_2_-glutamine labeling for 24 h. Schematic representation of the flux of isotopically labeled glutamine through representative metabolites from glutaminolysis, TCA cycle, and nucleotide biosynthesis; color-coded bars are showing intensities of different isotopes for each metabolite; white bars are reporting total pool intensities (average of three replicates). Error bars represent for standard deviations of total pool intensities (n=3).

C: control; A: AC220; I: IACS-010759; A&I: AC220-IACS
